# Hunterian Reminiscences; Being the Substance of a Course of Lectures on the Principles and Practice of Surgery

**Published:** 1834-01-01

**Authors:** 


*Hunterian Reminiscences; being the Substance of a Course of Lectures on the Principles and Practice of Surgery, delivered by the late* MR. JOHN HUNTER, *in the Year* 1785*: taken in Shorthand, and afterwards fairly transcribed, by the late* MR. JAMES PARKINSON, Author of "Organic Remains of a Former World." *Edited by his Son,* J. W. K. PARKINSON, M.R.C.S.L., *by whom are appended Illustrative Notes.*—London, 1833. 4to. pp. 176.

VOLUMES have been written on the genius of Hunter, orations have been spoken in his praise; but one glance round the gallery of his museum is worth them all. That was indeed a mind of no ordinary vigour and extent which could thus shadow forth, in one vast series, the universe of life. The imaginative grandeur of the conception was worthy of Milton; the labour of the work could have been surmounted by few men but John Hunter. Feeling towards his memory as all genuine lovers of science must feel, we have derived very high gratification from the perusal of these Reminiscences. It is no small pleasure to be able to place him as it were in his own person before us, delivering to his pupils those invaluable precepts which have since enabled many of them to labour so successfully in the same ample field: nor will we deny that even the peculiar and ungraceful style which pervades these, in common with all his writings, however objectionable in itself, still exerts a pleasing influence on our mind, as bearing the strong impress of his individual hand, and as the medium through which, troubled though it be, the most valuable doctrines of modern surgery have been transmitted to us. In some parts of these lectures, however, the language is much pleasanter, from being more familiar, than that which Mr. Hunter generally used; so that there is reason to regret that he has not always adopted a more colloquial manner of expression; for it is not to be denied that many have flung aside his works in disgust at the repulsiveness of their style.

Independently of its interest as a *relic*, this volume affords a valuable compendium of Hunter's principal doctrines both in physiology and pathology; the rather, that several of his opinions are here expressed more simply and intelligibly than in his other works. The following passage, for example, is worthy of particular attention, as completely vindicating its author from a charge to which, it must be confessed, his own extraordinary phraseology has very frequently exposed him,—the charge of absurdity in believing, like Van Helmont and Stahl, that the vital principle was a distinct being, and actually endowed with consciousness and intelligence.* We here find, however, that, so far from investing it with these imaginary attributes, he does not even assert its independent existence, but admits that it may depend on some arrangement of the particles of living matter, different from that which obtains in common matter. Now he tells us distinctly, in another place, (page 11,) that he regards the mind, and sensation, as the result of an arrangement in the brain and nerves, different from that which produces life: he cannot therefore mean to ascribe consciousness and intelligence, which imply mind and sensation, to the principle of simple life. His opinions respecting the mind we do not here intend to enter into. They evince, in common, we are sorry to add, with those of many other physiologists, a very

* Van Helmont believed that plants and minerals, as well as animals, had their intelligent living principle. There exists in all of them, it seems, a certain *aura*. "Quæ aura, licet in aliquibus luculentior sit: in vegetabilibus tamen succi specie comprimitur, ut et in metallis densissima homogeneitate inspissatur: singulis tamen hoc donum obtigit, quod Archeus vocatur, generationum et seminum fœcunditatem continens, tanquam causa efficiens interna. Ille inquam faber, generati imaginem habet, ad cujus initium, destinationes rerum agendarum componit. Constat Archeus verò ex connexione vitalis auræ, velut materiæ, cum imagine seminali, quæ est interior nucleus spiritualis, fœcunditatem seminis continens; est autem semen visibile hujus tantum siliqua. Imago hæc Archei, ex predecessoris idea defluens, vel eandem è condo externorum accipiens, non est demortuum quoddam simulacrum. sed plena insignitum scientia, potestatibusque necessariis, rerum, in sua destinatione agendarum ornatum; adeoque est vitæ, et sensationis primarium organum." (*Ortus Medicinæ*, ed. Amstelodami, 1652, p. 33.)

Those of our readers who are fond of the by-paths of medical literature will derive much amusement from the writings of this ingenious, but fanatical and conceited old Belgian.

imperfect acquaintance with the subject; which is the more to be lamented, since the force and ingenuity of some of his ideas on such themes shew how powerful a metaphysician he would have made, had his mind been properly trained to this kind of thinking. The basis, then, of Hunter's doctrine of vitality, is simply that living matter is subject to laws which do not influence common matter; an opinion which, we must say, in the midst of acknowledged uncertainty, appears to us to have common sense on its side.

"*Life.* Animal matter, then, is the result of a peculiar combination of other matter; and this combination or modification of common matter may be endowed with the living principle or not, the living principle not depending on that modification of matter which renders it animal matter; for this modification, which is peculiar to animal matter, and which constitutes it, may remain when the living principle is gone. Life is therefore something superadded to matter thus modified; or it is such an arrangement of the most minute particles of this modification as life may arise out of it, which arrangement may be the principle of life; and, if this arrangement be destroyed, the principle of life is also destroyed, and the part becomes dead animal matter; and, though it has suffered this alteration in its arrangement, and the loss of this principle, yet its modification, as far as our senses can discover, remains the same. This idea of life is hard to be illustrated; but magnetism perhaps affords the best illustration of it, for iron not magnetized may be considered as animal matter not endowed with life, and, when possessing the magnetic power, may be compared to animal matter possessing the vital principle: now, in neither of these can we discover by our senses, even when aided by art, any alteration in their modification when possessing or when without this principle. Light may, by its effects on bodies, yield us another illustration of this idea. This principle of life is the preserving principle of the animal, from its being the first cause of those actions whereby it is supported and continued. It is not sufficient that animal matter should be endowed with a principle of preservation; it must have action within itself; and the same arrangement which constitutes the principle of life does also constitute the principle of action: the *power of action* is indeed a step further. The most minute form possessing this necessary arrangement which our eyes can trace is the simple living fibre, whence is formed muscular fibres, muscles, organs, &c. There is this principle of action everywhere where there is life, for action is not confined to the muscles. Life I believe to exist in every part of an animal body, and to render it susceptible of impressions which excite action. This principle of action has been compared to the spring of a watch; but this comparison will not suit, since in a watch the spring alone possesses the principle of action, and is the immediate cause of action, and the action of all the other parts is entirely dependent on the impulses they receive from it; but, in the animal machine, every one will see that the principle of action resides in every part; so that, although action may be created through the whole animal by impulses from one part to the other, yet the principle of action is inherent in each of these parts, and independent of that of the others.

"The first and most simple idea of life is, its being a principle of self-preservation; the second is, its being a principle of action. The first of these may be conceived to exist without the other; it is not necessary that there should be action where we suppose the other principle to be. A fresh egg has no action, but is as much alive as a muscle: as a proof of which let us observe, that, when an egg is nearly hatched, the yolk is entirely sweet, as well as the albumen, notwithstanding the long heat it had been exposed to; but, if it does not hatch, it becomes putrid. And further:

"*Exp.* 1. A fresh egg was frozen, thawed again, and then exposed to the same degree of temperature (about the freezing-point,) with an entire fresh egg; and this latter, which had not been previously frozen and thawed, was six or seven minutes later in freezing than the former. This is to be accounted for by supposing that the former, having been deprived of its vital principle by the previous freezing, froze soon after its exposure to a degree of heat sufficiently low; but that the other egg, possessing the vital principle, resisted freezing until deprived of that principle.

"*Exp.* 2. An egg likewise which had been frozen, and then thawed, was frozen again in much less time than at first.

"*Exp.* 3. A fresh egg, and the egg which had been frozen, were put into a freezing mixture, at 15°, and a thermometer was placed in each: the heat of the dead egg came regularly down to 32°, where it directly froze; whereas the live egg came down to 29½°, where it remained fluid, and then rose again to 32°, where it directly froze. This is to be observed, that freezing may be prevented by many circumstances, and in this case by the presence of the living principle; and, where this happens, the fluid will contain heat some degrees below the freezing-point, and remain fluid until those particular circumstances no longer act, when it directly freezes, which happens in the present case until the living principle is destroyed. With respect to the principle of action, it is necessary that action should continue in some parts for the preservation even of the principle of action; but in other parts it is only necessary that the principle and power of action should remain, action itself not being in those parts constantly necessary. The living principle, we have before observed, is the principle of self-preservation in the animal, preserving it from putrefaction, which state soon comes on when life ceases, but much sooner in some instances than in others, appearing sometimes even before the departure of life: this last, we may suppose, is the consequence of a morbid or depraved action of the living principle, by which a putrefactive action takes place before death; but it is not certain whether in putrid fever, and even in the other instances, whether this alteration is not owing to some defect in the modification of the parts of the blood, and not a real putrefaction of the blood, which immediately before possessed their natural modification: at least, this process is different from the putrefactive process which takes place after death: nay, this very putrefaction being a part of the disease, the result of a morbid action, this process ceases when the actions of the animal are put a stop to by death, and then putrefaction, or the resolution of animal matter to common matter, begins to take place; so that we may say, that there are two kinds of putrefaction which may take place in animal matter; and it is observed that the latter putrefaction, after the death of such animals as have had this former process begun in them, has not proceeded faster on that account. I may here state, that I have seen two or three cases where this process had taken place before death, the cellular membrane being emphysematous with air thereby extricated; nor did the succeeding process of putrefaction appear to be so much quickened thereby as might have been expected. In the case of a man who had suffered the operation for aneurism of the popliteal artery, the leg, from about the middle of the calf downwards, became entirely cold, pale, and insensible, and was in every respect really dead, not being mortified, which is the consequence of a morbid action, but had as it were really died a natural death from having no blood sent into it. A few days after this man complained of being unwell, and in a few hours there were evident appearances of a resolution, or, if you please, a putrefaction through the whole living system, emphysema, opening of the cicatrix of the artery, &c.; but the leg, from the calf, suffered little or no alteration, and, after the man's death, it was put into the same degree of heat with the other leg."

We may here digress for a moment, to remark the extreme simplicity and beauty of Hunter's experiments, and the entire appositeness of his observations. It appears to us, that much of that power of discovering truth, for which he was so distinguished, resided in an extraordinary facility of invention, by which he saw, almost at a glance, the very experiment which would bring his notions to a satisfactory test. Placed as it were at the concourse of many ways, he would, as by a happy intuition, immediately plant his foot in that which led direct to the knowledge he wanted. Accordingly, we find a striking difference between his experiments and those of most other physiologists. The greater part of physiological experiments, however specious at first sight, have been found, after all, to prove nothing, or, if they prove anything, it is not the thing required; but each of Hunter's experiments was usually an *experimentum crucis*, and finally settled the point concerning which it was instituted: hence the greater part of those doctrines which he derived directly from experiment stand fast to the present day, and probably will do so to the end of time.

It was to this sagacity in eliciting facts by experiment, conjoined with an unwearied vigilance of mind, which converted even the apparently trifling occurrences of every-day life into the materials of thought, and the means of investigation, that Mr. Hunter appears to have owed his superiority, rather than to the extent or accuracy of his general powers as a reasoner. We may indeed observe a remarkable inequality in the operations of his mind. Where he can keep pretty close to the facts, his reasoning is generally correct and powerful; but where the subject is one which calls for lengthened deduction, it is often so much the reverse, that we can scarcely believe we are listening to the same man. And why was this? It was because he had never learned to reason. His natural endowments were of the very highest order, but they had never been regularly cultivated. It is often supposed that every man of great intellectual powers must necessarily be a good reasoner: this, however, is an error. Reasoning, like all other arts, has its fixed principles, without a knowledge and observance of which the most powerful mind on earth cannot practise it successfully in all its extent. It was the want of this knowledge in Mr. Hunter which has rendered some passages in his writings unintelligible; a defect which, we think, has been erroneously ascribed to insufficient command of language. The truth seems to be, that the reader cannot understand his meaning, because he did not understand it distinctly himself; the great activity and subtlety of his mind frequently leading him into abstract subjects which the defective discipline of his reasoning faculties did not enable him to grapple with. This source of obscurity in style is not peculiar to John Hunter; whenever a man's ideas are clearly defined to his own mind he can almost always render them intelligible to others:

"Verbaque provisam rem non invita sequentur."

He may express himself circuitously, clumsily, nay ungrammatically, yet he will be understood; when it is otherwise, the obscurity is in the thoughts rather than in the style. Accordingly, we find that Hunter himself, though his phraseology be generally cramped, and somewhat hard to make out, is still perfectly intelligible, except when his ideas get into a labyrinth. Of this misfortune the present work affords several examples, such as the observations on *diseased actions* and *altered actions*, some parts of which are altogether incomprehensible. It might be supposed that the transcriber had not been able accurately to catch the lecturer's meaning in such passages; but the fact is, all Mr. Hunter's works abound with similar instances.

We may here perhaps venture to suggest the importance of the study of dialectics to the student and practitioner of medicine. When we consider that the attention of the physician is continually occupied with the most abstruse and difficult questions, and that he is continually brought in contact, not only with the physical but with the moral and intellectual phenomena of man, is it not obvious that an accurate acquaintance with the principles of reasoning is essential to fit him for his office? And how is this to be attained without a familiarity with the nature and operations of the powers of the mind itself? Yet, as far as we have been able to observe, there is no class of educated men among whom these subjects are generally more neglected than the medical profession. In inculcating the necessity of this kind of knowledge, we cannot appeal to a stronger fact than that the want of it made such a man as John Hunter write many passages which nothing but respect for his name can rescue from the designation of nonsense!

Had Hunter been early trained in habits of severe reasoning, his would have been perhaps the most majestic intellect that ever shed its light over the field of science: even as it was, how great were the results of his untutored genius!

These lectures are not to be considered strictly as a course of surgery; they are rather an application of general physiological and pathological views to surgical subjects: for Mr. Hunter well knew that he could occupy the time of his pupils much more profitably than by the details of those mechanical parts of the art, which they could learn for themselves much better in the dissecting-room than any lecturer could teach them.

Before entering on the surgical portion of the work, we cannot refrain from transcribing the following highly interesting observations on Delirium.

"It will be very difficult to prove whether delirium is a disease of the brain or nerves. From the connexion between dreams and delirium, it will be necessary to consider sleep and dreams. Perfect sleep is a cessation of the susceptibility of sensation, a cessation of the consciousness of existence, and a cessation of the relationship which our bodies bear with other bodies; but whether this cessation of susceptibility arises from the brain not having the power of receiving, or the nerves not having the power of conveying, is yet to be determined. Dreams proceed from an action of the mind in sleep, and therefore may be independent of impression, and are without a consciousness of the relationship between the mind and body, and the body and other bodies; therefore, in dreams, we lose that power of distinguishing between real sensation and thought, which constitutes wakefulness. In a delirium, as in sleep, we find the susceptibility of external impression is lessened. Whilst sensation is continued sleep is kept off: delirium likewise may be lessened, by arousing the mind from that particular state by external impressions; so far delirium appears similar to dreams, but it widely differs in other respects. In natural sleep, the more the brain puts on that peculiar state, the *less* we have of dreaming; but the more the other state is put on, the *greater* the delirium. Dreams often do arise from sensations of the body being conveyed to the brain, it being an imperfect sleep; but the consciousness of the connexion between our own body and our own mind being cut off by the state of sleep, the sensation may, or may not, be referred to our own body; it may be referred to some other body. In some cases it is not referred to the part of the body whence the impression is received: the same thing happens in delirium, when the connexion is cut off; then, not distinguishing between real sensation and thought, what the mind thinks about appears to be real. But even where the mind is in full possession of the consciousness of its connexion and relationship with the body, we have in some cases this delusion, as the appearance of the turning round of the objects around us, whilst they are really fixed, and that in consequence of our having turned round quickly; giddiness from going to a height; from riding backwards in a coach: delusion is also an effect of intoxication and disease. Whilst awake, and in health, impressions produce sensations which are conveyed to the brain, and from these the mind reasons; but, suppose the mind to have lost, or as it were forgot its former connexion with our body, then the above false reference takes place.

"CASE I. A gentleman came into this country in 17—; his memory was imperfect, and a particular kind of delirium began whenever he was going to sleep, but afterwards continued whilst wide awake, and for a week before his death he was not quiet from this delirium a moment, but whilst impressions were forced on him by external objects. His delirium was of this kind: he was continually talking of former circumstances of his life, but referring them to the present moment, and to some other person. There was a revival of past ideas in his mind; but, from want of connexion between his mind and body, he was not enabled, by his present impressions, to infer how little relationship they bore to the present time, or to those persons to whom he referred them; at the same time it really appeared more a want of connexion between the mind and body than the mind itself being hurt, for he determined rightly what should be done in those circumstances which he supposed present, and would express his sentiments in really elegant language. That it depended more on a want of connexion than on disease of the body, appeared from his being sensible of impressions, and referring them to the part where they took place, but supposing that to be in any other body but his own; thus he would tell his nurse and the bystanders that they were hungry, or thirsty; but upon offering food or drink it appeared plainly, by his eagerness, that the idea had arisen from a sensation of hunger in his own stomach. He would shew great signs of distress and anxiety, which, he would say, was because his nurse wanted to go to the close-stool, but was restrained by his presence, and this from his own sensations also; he had a violent cough, which he would sympathise with some bystander in, proceeding in his story after the cough, no otherwise disturbed than by sympathizing with the person whom he thought so unfortunate in having it. The objects about him were more to him than his own sensations.

"CASE II. A gentleman, who was fond of his bottle, as he became intoxicated, referred all his own weaknesses and feelings to those around him, supposing that every one but himself was drunk; and upon his going home would insist upon undressing all his family, and putting them to bed, declaring that they were too drunk to do it themselves; and this happened not once only, but whenever he was intoxicated. I myself once experienced what I have since thought must have proceeded from this want of connexion between the mind and body. I was reading a remarkable case, and reasoning with myself upon it, when I found the letters and words made an impression on the retina, but that I was incapable of affixing a meaning to them; this, I thought, might proceed from want of sleep, but that was not the case. I tried repeatedly, but without effect; and at last went to bed, from which I did not move for three weeks, a violent complaint in the head succeeding this extraordinary circumstance. It may not be amiss to say, that the case I was reading was that of the late Mr. Foote, who was not able to command his attention to more than one circumstance or action at a time; thus, if he took his snuff-box out of his pocket, and held it in his hand, it was very well, until he attempted another action, such as taking a pinch of snuff out of it, and then the box immediately fell out of his hand: in fact, he was going back into a state of childhood; for a child is not capable of commanding his attention to more than one circumstance or action at a time: give him first a stick to hold, and call his attention to another object, the stick will be dropped; for it is by habit we become capable of attending to several actions at a time. Turning round, or passing quickly by different objects, it appears as if they were in rapid motion; and if the motion of the body is stopped, the delusion will continue for some time. If a person is blindfolded, and put into a coach, he will think he is moving forwards, though he is really riding backwards. An impression from any part, either healthy or diseased, may be conveyed, the mind having full possession of the impression, and a perfect idea of it, but having nothing to direct it right in its reference of it. It must refer it somewhere, and is more likely to refer to another than to itself."

4

"CASE III. A gentleman (a medical man) dreamed he had given to a patient too strong an injection, for a gonorrhœa, and that it had produced a total stoppage of urine: he awoke, and found an erection of the corpus cavernosum of his own penis, and that he could not void a drop of urine. Here was impression without the consciousness, and hence he referred his own feelings to another person.

"CASE IV. A gentleman upwards of ninety years of age suddenly lost his senses, and, in consequence of this, was a reference of all the ails which he might be supposed to feel to his wife, who had been dead some time, but whom he now thought alive, and ordering the utmost silence to be observed, lest by noise her illness should be increased. The new-born child has probably sensation without this consciousness. The contrary takes place where a person refers the sensation of others to themselves, or where the idea of sensation is supposed to be sensation itself, as happens to those who are affected by animal magnetism. I was asked to go to be magnetized, but at first refused, because the spasm on my vital parts was very likely to be brought on by a state of mind anxious about any event. Thus, at my country-box, I have bees, which I am very fond of, and I once was anxious about their swarming, lest it should not happen before I set off for town; this brought it on. The cats tease me very much, by destroying my tame pheasants, partridges, &c., and rooting up my plants. I saw a large cat sitting at the root of a tree, and was going into the house for a gun, when I became anxious lest she should get away before my return; this likewise brought on the spasm: other states, where my mind is much more affected, will not bring it on. Now I feared lest my anxiety for the event should bring on my spasm, and that should be imputed to animal magnetism; but, considering that if any person was affected by it, it must be by the imagination being worked up by attention to the part expected to be affected, and thinking I could counteract this, I went: and, accordingly, when I arrived at the place, I was convinced, by the apparatus, that everything was calculated to affect the imagination. When the magnetizer began his operations, and informed me that I should feel it first at the roots of the nails of that hand nearest the apparatus, I fixed my attention on my great toe, where I was wishing to have a fit of the gout; and I am confident that I can fix my attention to any part, until I feel a sensation in that part. Whenever I found myself attending to his tricks, I fell to work with my great toe, working it about, &c. by which means I prevented its having any effect on me."

The concluding anecdote is very characteristic of Mr. Hunter, and we recommend it to the especial attention of the magnetists of the day; for whose further edification we may relate a curious circumstance which occurred to us some months ago. On our applying the stethoscope to the chest of a poor and very ignorant patient, she suddenly gave a deep groan, and fell back in a state approaching to syncope: on being asked what was the matter, she said that the instrument *drew* her too strongly. She had supposed that it was to act as a remedial agent, probably by drawing the disease out of her; and she hence referred an imaginary feeling to the place of its application, or possibly exaggerated the slight uneasiness occasioned by its pressure into an overwhelming sensation. Now, had the stethoscope been a magnetical apparatus, and we professors of the art, this might have been set down as a striking case. It is by "facts" such as this that the pseudo-sciences delude their votaries.

The remarks on *Inflammation* afford a very useful summary of the author's great work on that subject; but, as we hope that its contents are engraved on the memory of every British surgeon, we shall not dwell upon them here. Among the miscellaneous subjects afterwards treated of, the following observations on *Hemorrhage* will be read with interest:

"Discharge of blood may be natural, or it may arise from disease or accident. Of the first kind is the menstrual discharge; of the second, are discharges of blood from the nose, lungs, intestines, piles, excessive menstruation, &c. These may be local or constitutional; but the local are more to our present purpose: the causes may be either a peculiar irritation, or a species of relaxation in the vessels of the part. With respect to bleeding from the first cause, that is not a very unfrequent circumstance, the vessels of the part being affected by some particular irritation: bleeding from this cause I have frequently seen upon the action of a new stimulus; thus, I have noticed, after many operations, when the bleeding had stopped by the natural contraction of the arteries, that they have opened again upon the application of the actual cautery in their neighbourhood. A discharge of blood from this cause will sometimes happen from surfaces, which otherwise might have been expected to discharge pus. In these cases, instead of the application of styptics, as they are called, sedatives should be applied: the best of these are perhaps opium, and the different preparations of lead. I was called to a sore, which was discharging great quantities of blood; but this was stopped by a poultice of poppy-heads. The loss of no fluid has such effects on the system as that of blood, producing faintings, coldness, cold sweats, &c. Where the loss has been considerable, the absence of the pulse, and extreme coldness, shew the patient to be near the last extremity, and death indeed would most probably be the consequence of any farther loss of blood; but it generally happens that these alarming symptoms immediately following the bleeding are succeeded by a feverish heat, with a quick strong pulse; and here it has been considered whether bleeding is not necessary to prevent a return of the disease: but it is my opinion, that this rising of the pulse and return of heat are in consequence of a struggle of nature from the loss she has sustained, as if calling up every power. Secondly, hæmorrhages, from relaxation, or want of disposition to contract, may happen from external force applied, or from disease. The mouths of the vessels may be stopped; first, by the natural muscular contraction of the cut edges of the vessel, the stimulus from the accident producing this contraction, so that, in this case, the cure arises out of the accident. I do not think if the arteries below the knee were divided by amputation, that the patient would be killed before the natural contraction of the arteries took place. The bleeding was stopped by the natural contraction of the artery in a boar whose thigh was amputated; and in an ass who had bled to the death from these arteries, the artery was, after death, found closely contracted; but I believe the arteries of quadrupeds to be more contractile than ours. This contraction is the consequence of the stimulus from exposure and division. Leeches make wounds, for their size, the most difficult to stop of any: this has been said to have been from their taking a piece out of the side of the vessel: if this were all, the contraction of the vessel would be sufficient to stop the bleeding; but I am more inclined to think that the leech poisons the vessel, in consequence of which its contractility is destroyed. Oil of turpentine is perhaps one of the best styptics: it should be applied to the wound, the wound being first wiped clean, and the bleeding checked by pressure, that it may be fairly applied. I have given it often with great success internally, when other things had failed. A gentleman who had had repeated bleedings at his nose, until he appeared like a corpse, sent for me upon a return of his bleeding. I ordered him a draught every two or three hours, with ten drops of oil of turpentine made up with yolk of egg: this stopped the bleeding, and he has continued well ever since; for, whenever there is the least disposition to a return of the bleeding, he takes a draught, which he says prevents it. Another mode by which bleeding is stopped is, by pressure of the coagulated blood round the mouth of the vessel; this is chiefly effectual where the orifice is very large; acting by pressure, it is aided by spongy substances being applied, as lint, agaric, &c. The lateral wounds of arteries are commonly, though improperly, termed false aneurisms; and this has probably originated from a pulsation being felt in them, it having been supposed that only the external coat of the artery had been wounded, and that the internal had given way at this place, having lost the support of its external coat: but the fact is, that the blood escapes into the cellular membrane, and, assisting the external pressure, may retard the bleeding; and where speedy dressings are applied, the bleeding may for the present be stopped; nay, the external wound may be healed by the first intention; but, by the constant and increasing pressure, the surrounding cellular membrane that contains the extravasated blood by degrees gives way, and forms a perfect cyst. These may be divided into two stages: first, whilst the case is yet recent; secondly, where the cyst is formed. The artery should be taken up before the cyst is formed.

"CASE. A young man wounded himself in the thigh; both the crural vein and artery were obliged to be taken up. I would in cases of wounded artery, in bleeding, give a chance to the artery of healing, by speedy dressing, pressure, &c. I made a small puncture in the crural artery of a dog, which healed, and no aneurism was formed."

The subject of *Traumatic Tetanus* is one on which we are still so much benighted, that the sentiments of all intelligent men are worthy of an attentive hearing; we therefore give at full length those of Mr. Hunter, whose military practice must have afforded him considerable opportunities of witnessing the disease, and whose accurate observation and deep reflection render all his opinions on practical points so valuable. Unfortunately, all experience up to the present time does but confirm, with respect to this, the judgment which Hippocrates gave on all convulsive affections supervening on wounds: Επὶ τρώματι σπασμὸς ἐπιγενόμενος θανάσιμον, (*Aphorism.* lib. iv. sect. v. 2.); a maxim, to the general truth of which all experienced surgeons can testify.

*Locked Jaw.* "This may be considered as a partial effect of tetanus, and generally the beginning. When the muscles on the fore part of the trunk are affected, it is termed emprosthotonos; when on the back part, opisthotonos. They may be classed with the unnatural or involuntary contractions. Of these is formed a genus, of which tetanus and locked jaw is a species, and of which the subsultus tendinum, spasmus cygnicus, wry-neck, &c. are lower species. Horses, cows, deer, monkeys, and other animals, are liable to this disease. A stag I had died of it. Some parts are more disposed to these affections than others are, as the muscles of the lower jaw; the order of muscles next disposed to it are those which are nearest. The disease spreads, by sympathy, I suppose, through the different muscles. Its cause and nature have not hitherto been sufficiently attended to. Having followed wounds very frequently, it has been supposed a consequence of irritation from those wounds; but these wounds do not comprise the whole of the cause, for irritation merely, such as tends to produce inflammation, will not produce it. A slight irritation may be the exciting cause; but, as a predisposing cause, there must, I think, exist a weak and irritable habit. We seldom find it in a good consitution, or accompanying inflammation. Where there is inflammation, it is always previous to the spasm, the latter coming on generally when the inflammation goes off. This disease often happens from very trifling exciting causes; yet we are not to confine ourselves to these, but likewise attend to the predisposing cause, or the disposition in the habit for such actions. It is not the consequence of irritation increasing the action of the living principle, as in inflammation, but an irritation of the nervous principle; or, in other words, the disposition consists in irritability of the nervous system, such as is possessed by those who have a proneness to nervous complaints, not an irritability disposing to inflammatory symptoms. In such a disposition it is my idea that the irritation of a trifling wound (and perhaps from some peculiarity in the irritation,) may be the immediate cause, even though the irritation may not amount to the height of pain; and this irritation may have more effect than a greater degree of pain: thus, tickling may have a greater effect on the nerves than a violent blow on the part. An injury will have a greater effect on the minds of those of weak constitutions than on the strong. Weakened constitutions, we know, are always prone to what are called nervous symptoms. In a woman with child the nervous principle is weakened, though the living principle may be very strong, and exhibits an instance of this kind of constitution.

"CASE. I once threw a stone at a deer with so considerable an exertion as to produce an extravasation of blood in the arm; the muscles were so affected, that a loss of motion in the arm immediately followed. I felt myself exceedingly weak, and was very pale; after a little time cramp came on in my leg, but at last this and the other symptoms went gradually off: if the irritation had continued, a locked jaw might have come on. Now here, from the irritation arising from the injury done to the arm, a spasmodic affection was produced in a person immediately before in perfect health.

"This complaint is most frequent in warm climates, and is even very rarely found in cold ones. Preceding inflammation may be sometimes a predisposing cause, by rendering the nervous system more irritable. Wounds of tendons, ligaments, &c. are likely to be the immediate cause, from their possessing so very slight a power of healing, and thereby keeping up a more constant irritation on the nervous system. How much a wound in such a part weakens, may be deduced from observing that the limb wastes when such a wound has existed any considerable time.

"The wounds producing this disease may be supposed either considerable or slight: the first seems to act as a predisposing cause as well as immediate; the latter, as immediate only. In the first case, the patient is not attacked with the locked jaw until the inflammation is nearly gone off; in general, not until a good suppuration has come on; nay, in some I have seen the healing of the wound so far advanced as to be completed before the jaw unlocked; whereas, in those from slight causes, the locked jaw comes on before the wound begins to heal; and in these cases the disposition may be supposed to be already subsisting. It much oftener appears as the consequence of a slight wound than of a considerable one. It may arise in consequence of other weakening causes, as fever, flux, &c. The period at which it becomes dangerous, by attacking vital parts, seems to be before the constitution has become habituated to it; this is generally within a fortnight: if it does not kill within the first fortnight, the patient generally recovers, and that even though the symptoms continue violent some time afterwards. It kills by the disposition increasing, and the effects extending until they reach some vital part, and which it affects with spasm: in this manner also I think the gout kills.

"The cure has hitherto been attempted by everything which can be termed antispasmodic, but with very little success: the one which has kept its ground longest is opium, though this, in my opinion, has not done the good that was supposed: the cases would, most probably, have done well without it. It may lessen the effects, but cannot remove the cause; it may prevent the simple sensation of pain in the mind, and may do good so far by preventing the pain from weakening still further the patient. Electricity seems to relax the spasm at the moment, but it soon comes on again. Actual cold seems to be the most efficacious remedy, and may be applied to the whole body, or topically to the part. If I were to have a locked jaw, I would be put in an icehouse; the patient would do well therefore to remove to a colder climate.

"Internal medicines I know of none. Forcing the mouth open seems sometimes to do good, but I was told it once killed a patient. In one case, where bleeding was performed about the tenth day of the disease, the patient fainted away, became immediately worse, and died soon after. In another case there seemed to be but little connexion between the wound and the locked jaw; the wound healed, and a considerable time before the jaw unlocked. Opium and musk were both tried in this case, without any effect. In another case, two grains of opium were given every half hour, and increased to three grains every quarter of an hour, with no good effect. Volatile medicines were also given freely in this case. The spasm continued even a short time after death. In another case, the patient, who was quite bent backwards, was put into the cold bath, and was relieved a little whilst in the bath, but was as bad as ever when taken out: it was twice repeated with the same relief; but the moving him put him to so much pain, that he would not allow it to be again repeated. Another patient was so much relieved, when the trunk and jaw was quite stiff, as to be found the next day sitting on the side of the bed. Being obliged to embark for Portugal, I left the patient in this state with the surgeon."

The opinions of surgeons in the present day generally coincide with that of Mr. Hunter as to the nervous character of tetanus: there exists, however, one exception, to which it is of great importance to attend. All the symptoms of tetanus occasionally supervene on inflammation of the spinal chord, (*Abercrombie*, in *Edin. Med. and Surg. Journal*, vol. xiv.; *Ollivier, Traité de la Moelle épinière*, p. 705, *et alibi,)* in which case the stimulant treatment most generally adopted, and perhaps most likely to be serviceable, where the disease arises primarily from nervous irritation, would be in the highest degree injurious; and the only chance of removing the tetanic symptoms is by resolving the inflammatory affection on which they depend.

The following remarks on *Fungated Sores* are highly interesting, as they render it probable that the disease now familiarly known to surgeons under the name of Fungus Hæmatodes, and believed to have been first accurately described by Dr. J. Burns under that of Spongoid Inflammation, had not escaped the ever-watchful observation of Mr. Hunter.

"I mean now to speak of what I call fungated sores, and which are often taken for cancers; but they agree with cancer, I believe, in nothing but in being incurable. There is one very striking difference between these diseases,—the cancer appears to eat the parts away, whilst in this new parts are added. This disease appears to me a specific one, and a well-marked disease. It seems to be common to every part of the body, while I believe cancer is not. The beginning of this disease is, I think, generally in the form of a tumour, either solid or encysted. As soon as the skin gives way, fungi arise, of a very dark colour: this rises so fast, that I believe we have no escharotic which can keep pace with it. It yields a plentiful discharge of matter, but which does not appear to be a poison, as I never saw the lymphatics affected by it. This is the foot of a patient I had in St. George's Hospital. You see from the sole of the foot what a considerable quantity of this dark fungus arises. In this case there was no disease of the lymphatics proceeding from the foot, but, about a fortnight after the amputation, the stump swelled amazingly: that however went off, and the patient mended very fast. Here you may observe the same kind of fungus arising from the testicle: here was no alteration in the spermatic chord. I stuck into the substance of this fungus troches of arsenic and wheat-flour, but these did not seem to produce any effect on it. It is this disease also which is the cause of that excessive enlargement of the penis that is generally supposed to be cancerous: this is generally harder than the common fungus of fungated ulcer. The surface of this fungus is in general tolerably smooth, and, when cut into, it seems to have somewhat both of a striated and radiated appearance in its substance.

"As there are no means yet discovered by which it can be made to take on a healing disposition, there is nothing left for us but a strict extirpation, so as to leave no part behind; if, in the extremity, amputation is most proper. It is worthy of notice, that this disease kills with much less visible cause of mischief than cancer. The first case I saw of this, arising from accident, was in an ostler at the Dun Horse, in the Borough, who had received a kick from a horse on his fore-arm. This broke into an ulcer of this fungated kind, and his arm was amputated."

It is possible that more than one disease may be here delineated; but the description of a tumour which at last ulcerates, and sends forth a fungus of large size and rapid growth, the circumstance of its attacking all parts of the body, and the universal fatality of the disease, can hardly be made to apply to any other affection than fungus hæmatodes.

While on this subject, we may remark, in passing, that common fungous ulcers are generally treated much too lightly in surgical works. Fungous granulations are frequently spoken of as a very simple matter, to be got rid of by a little sulphate of copper, and no doubt they often are so; but, in cases of old ulcers, which had never hitherto presented any malignant character, we have seen what at first appeared to be nothing but exuberant granulations from their surface gradually increase in size, in spite of pressure and escharotic applications, till at last they have formed a large tumour, of a soft texture and exceedingly rapid growth, scarcely distinguishable, in any of its features, from fungus hæmatodes, and which ultimately destroyed the patient by its effects on the constitution.

In concluding our extracts, we have to return our best thanks to Mr. Parkinson for the pleasure his publication has afforded us. He has acquitted himself creditably in his capacity of editor, by a well-written preface, and some ingenious notes; and we strongly recommend these Reminiscences to the attention of the profession, to whom, we are sure, they will not be the less acceptable because taken down by the hand of the late learned author of the "Organic Remains," who has conferred such large obligations on the scientific literature of his country.

